# Web-Based Health Resources at US Colleges: Early Patterns and Missed Opportunities in Preventive Health

**Published:** 2011-10-15

**Authors:** J. Jane S. Jue, Joshua P. Metlay

**Affiliations:** ECRI Institute. At the time of this study, Dr Jue was affiliated with the Department of Veterans Affairs, Philadelphia VA Medical Center in Philadelphia, Pennsylvania, and with the Robert Wood Johnson Foundation Clinical Scholars Program at the University of Pennsylvania School of Medicine in Philadelphia, Pennsylvania; University of Pennsylvania School of Medicine, Philadelphia, Pennsylvania. Dr Metlay is also affiliated with the Robert Wood Johnson Foundation Clinical Scholars Program, Philadelphia, Pennsylvania

## Abstract

**Introduction:**

Web-based health resources on college websites have the potential to reach a substantial number of college students. The objective of this study was to characterize how colleges use their websites to educate about and promote health.

**Methods:**

This study was a cross-sectional analysis of websites from a nationally representative sample of 426 US colleges. Reviewers abstracted information about Web-based health resources from college websites, namely health information, Web links to outside health resources, and interactive Web-based health programs.

**Results:**

Nearly 60% of US colleges provided health resources on their websites, 49% provided health information, 48% provided links to outside resources, and 28% provided interactive Web-based health programs. The most common topics of Web-based health resources were mental health and general health.

**Conclusion:**

We found widespread presence of Web-based health resources available from various delivery modes and covering a range of health topics. Although further research in this new modality is warranted, Web-based health resources hold promise for reaching more US college students.

## Introduction

In 2008, more than 18 million people in the United States were enrolled in college (1), most of whom were young adults aged 18 to 25 years. These young adults are in a unique developmental stage, transitioning to autonomy in decision making and independently developing behavior patterns (2), in particular health behaviors, that they will often continue throughout their lives (3).

Health and disease prevention are often not a priority of young adults. However, when seeking health information, they most often use the Internet, citing accessibility, availability, privacy, and confidentiality as reasons for preference over traditional sources (4). They most often use search engines to seek out health information online (5,6), even though this is not the most efficient way to access health information (7). Young adults experience difficulty in assessing the reliability and quality of information found online (4,8-10). In 1 survey, 90% of college students found college medical center staff to be a credible source for health information compared with other sources (4). No study looks specifically at youth attitudes and behaviors around Web-based health resources on college websites. Yet, given that college student health seeking occurs primarily on the Internet and the most believable source of health information is perceived to be local student health center staff, providing health resources on local college websites may be an effective way to educate and promote health in young adults.

The objective of this study was to characterize how colleges use their websites to educate about and promote health. We describe the presence of various Web-based health resources on college websites. We assessed both the breadth of health categories covered and also the variety of Web-based modes of delivery, which included Web-based health information, links to outside health resources, and interactive Web-based health programs. As a secondary objective, we investigated the hypotheses that the size of the college, public versus private school status, health professional school affiliations, and presence of student health services and health professionals increase the likelihood of health resources being present on college websites.

## Methods

### Study sample

We conducted a cross-sectional analysis of websites from a nationally representative sample of US colleges. Eligibility criteria included 1) being an accredited 2-year or 4-year bachelors, associate, or trade school degree-granting institution; 2) having at least 1 physical campus located in the United States; 3) having an institutional website; and 4) currently enrolling students. We excluded colleges that were online only, institutions that were graduate-level only, and colleges whose websites were not accessible (ie, password protected). The colleges were selected from the 2009 Higher Education Directory, a comprehensive database of all higher education institutions in the United States (Higher Education Publications, Reston, Virginia). Twelve strata were created based on 1) geographic region (Northeast, South, Midwest, and West) and 2) student body size, including graduate students where applicable (small, <5,000; medium, 5,000-9,999; large, ≥10,000). We randomly selected 30 to 33 colleges from each strata (n = 385 total). We also oversampled 4-year colleges, randomly selecting 8 to 10 four-year colleges from the 12 strata (N = 100). Thus, the final sample included a total of 485 colleges out of a total of 3,506 eligible institutions. Of the 485 colleges, 59 were excluded, and 426 eligible colleges were included in the final sample.

### Data collection

From February 1, 2009, through April 30, 2009, 2 reviewers (ie, abstractors) abstracted data from college websites by using a standardized abstraction tool (Appendix A). Abstractors visited colleges' official websites as listed in the 2009 Higher Education Directory. In addition to the college main website, reviewers also abstracted data from college student health services and counseling websites (when available). Abstractors were instructed to search for key terms (Appendix B) and look through relevant links from both the main college website and the student health services websites, if applicable. All data collected were in the domain of the college website and did not include student personal or nonaffiliated student group websites. There was no limit to the number of separate websites that could be visited for data collection from each college. To assess interrater reliability, the 2 reviewers both abstracted a 10% overlap of websites. Interrater agreement between abstractors was assessed, and we calculated the Κ statistic for the presence of health information and interactive Web-based health programs (Κ = 0.8 for both). A third reviewer resolved discrepancies between the 2 primary reviewers by examining the websites of those colleges. We held regular meetings with reviewers to discuss questions, issues, and discrepancies.

### Web-based health resources

The primary measure of interest was the presence of Web-based health resources on college websites. The study was designed to assess both the breadth of health categories covered and also the variety of Web-based modes of delivery. We identified 4 major health categories: 1) general health, 2) reproductive and sexual health, 3) substance abuse, and 4) mental health. Each health category was further subdivided into specific content areas (eg, asthma, depression). The categories and content area in each were based in part on the critical health objectives (11) for young adults determined by the Centers for Disease Control and Prevention (CDC) as part of *Healthy People 2010* (12). Also identified were 3 Web-based health delivery modes, including 1) direct health information provided directly on websites, 2) outside Web links to other health-related websites, and 3) interactive Web-based health programs. *Direct* *health information* was defined as specific information about a health topic or disease that detailed content such as epidemiology of the illness, symptoms, diagnosis, and treatment. Simple listing of the availability of illness-specific services provided did not count as health information. For example, a website describing signs, symptoms, and treatment of chlamydia infections would be considered health information. However, a website merely stating that their student health clinic provided services for chlamydia did not count as providing health information. *Outside Web links* were defined as Internet links that brought a user from the college website to a noncollege website that provided health information or health-related resources. An *interactive Web-based health program* was defined as an interactive program accessed on the Internet that addressed a health topic. Though interactive Web-based health programs are neither exclusive of nor necessarily a subcategory of health information, significant overlap exists. The difference from health information was the interactive component, such as an online assessment, a program or file download (eg, podcast), or assessments that were evaluated (eg, submit a survey by e-mail for evaluation).

### Institutional characteristics

We collected data on only institutional characteristics as reported in the 2009 Higher Education Directory; these were school name, location, website address, enrollment size, 2-year versus 4-year college, and public versus private status. We collected data on additional institutional characteristics from the college website; these included the presence or absence of campus student health services, counseling services, and staffing, including nurses, health educators, midlevel providers, counselors, and physicians, and affiliations with medical, nursing, and public health schools or programs. Student health services and counseling services are health services or centers focused on students of that college. They need not be physically on campus, but they must be more than an affiliation with or referral to outside health care providers. We determined staffing at student health services and counseling services from website listings on the basis of title and degree.

### Statistical analysis

To generate a final data sample reflective of the population of eligible US colleges, all analyses were weighted by the inverse sampling probability for each sampling strata. The weighting accounted for both the strata and the oversampling. We calculated descriptive statistics of the sample and prevalence of each health resource along with binomial confidence limits. All analyses were conducted by using Stata version 10 (StataCorp College Station, Texas). To assess the association between institutional characteristics and the presence of each Web-based health resource by health category, we conducted weighted logistic regressions by using the Stata survey command. The outcome variables were the presence versus absence of various health categories of Web-based health resources (eg, Web-based resources on mental health), while the predictor variables were the institutional characteristics (eg, presence of student health services). We created final adjusted models by using all institutional characteristics and backward selection (*P* < .1) to identify independent predictors for each of the 4 health categories and also each of the 3 delivery modes. A priori, we decided to force certain institutional characteristics into the model because we believed they were important, including public or private status, 2-year or 4-year status, and enrollment size. The study was determined to be exempt from institutional review by the human subjects subcommittee at the University of Pennsylvania.

## Results

### College characteristics

Most colleges were public, small (<5,000 students), and 4-year, findings that were consistent with other estimates (Table 1). We derived weighted characteristics of US colleges from the study sample. Most (77%) colleges had some form of student health services available, with a range of health care providers listed on staff.

### Use of Web-based health resources by US colleges

Colleges with health educators, 4-year colleges, and large colleges were more likely to provide Web-based health resources in all health categories than were 2-year colleges (Table 2). More colleges provided direct health information in general health (24%), followed by mental health (18%) (Table 3). Of all colleges, 58% provided at least 1 of the 3 modes of delivery of Web-based health resources (direct health information, interactive Web-based programs, and outside links) on their websites for any health topic. Specifically on their websites, 49% provided health information, 48% provided links to outside health resources, and 28% provided interactive Web-based health programs. Sixty-eight percent of colleges with student health services, compared with 22% of colleges without student health services, had some Web-based health resources. Of those providing any Web-based health resources, 76% covered general health topics, 55% covered reproductive and sexual health, 65% covered substance abuse, and 82% covered mental health topics.

### Links to outside health resources

Overall, 48% of colleges provided Web links to outside health resources. Mental health (36%) and general health (36%) were provided most often (Figure). In each health category, links to outside health resources were more prevalent than either direct health information or interactive Web-based health programs provided on college websites. Of the 51% of colleges that did not provide health information directly on their websites, only 17% provided links to outside resources as an alternative. However, of those that did not provide direct health information on mental health, 31% provided links to outside mental health resources.

**Figure.**
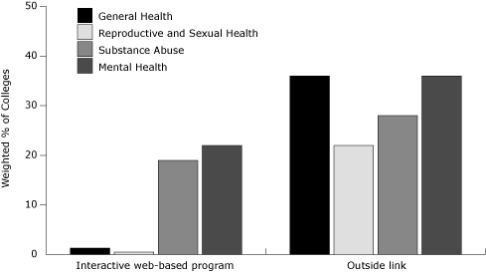
Percentage of US colleges with Web-based health resources, by delivery mode and category, February-April, 2009.

**Table:** 

**Delivery Mode**	**General Health**	**Reproductive and Sexual Health**	**Substance Abuse**	**Mental Health**
Outside link	36	22	28	36
Interactive web program	1.3	0.5	19	22

### Interactive Web-based health programs

The highest percentages of interactive Web-based health programs were in the areas of mental health (22%) and substance abuse (19%). The most common interactive Web-based health programs for mental health were for stress and anxiety (93%). For substance abuse, the most common were for alcohol use (30%). Tobacco use cessation via interactive Web-based health programs was observed at only 1.2% of colleges overall. Similarly, interactive Web-based health programs in the areas of reproductive health (0.5%) and nutrition and weight management (0.4%) were identified in few college websites.

## Discussion

### Early patterns: mental health resources are prominent

The breadth of health information covered varied substantially by college, the highest percentage of schools providing information for any specific, single topic was only 14.8%. More colleges provided links to outside health resources than provided direct Web-based health information or interactive Web-based health programs on their websites. Interactive Web-based health programs were notably absent for general health and sexual and reproductive health topics and more prevalent for substance abuse and mental health topics.

Mental health had the most overall Web-based health resources on college websites and was the topical category with the most interactive Web-based health programs, specifically programs addressing stress and depression. These findings may reflect both an increase in mental health needs in general and a growing recognition of the need to address behavioral health in young adults. Studies show that though approximately 15% of college students either report a diagnosis of or have symptoms consistent with a diagnosis of depression (13,14), most do not receive treatment (15). Web-based mental health programs may be particularly appealing to young adults, as they help overcome barriers to treating mental health: stigma and cost. Such programs can also decrease the financial burden on colleges for providing information, skill-building, and screening for mental health problems. Another reason that may explain the high number of interactive Web-based health programs in mental health may be related to market forces. One predominant interactive Web-based mental health program that was sponsored by a suicide prevention foundation provided customized interactive Web-based mental health programs to colleges at no cost.

### Missed opportunities: tobacco use, nutrition, and physical activity

In addition to early prominent patterns, there were also notable absences among particular topical areas. Interactive Web-based health programs addressing tobacco use cessation were uncommon, despite tobacco use being the leading cause of preventable death in the United States (16). Most smokers start before the age of 18 (17), and many arrive at college already addicted to cigarette smoking. However, younger smokers (aged 18-24 y) compared with older smokers (aged 35-64 y) and those who are college educated compared with those who are not are more likely to quit (18,19). Despite this crucial period to target behavior change, less than 10% of colleges in this study offered health information and only 1.5% of colleges offered interactive Web-based health programs related to tobacco use cessation on their websites.

The second leading cause of preventable death in the United States is overweight and obesity due to improper diet and physical inactivity (16). Young adults are the age group at highest risk for weight gain (20-23). Studies also find that when using the Internet to obtain health-related information, adolescents and college students most often seek it in the areas of fitness, exercise, diet, and nutrition (6,8). However, fewer than 10% of colleges provided Web-based health resources related to nutrition or physical activity, and very few nutrition-related interactive Web-based health programs were provided.

The lack of Web-based health resources in tobacco use cessation, nutrition, physical activity, and weight management represent missed opportunities in the prevention of chronic diseases of adulthood. These preventable illnesses are relevant to young adults during the period when many health behaviors are solidified. Targeting behaviors among college students by using Web-based health resources may be a promising avenue to tackle 2 of CDC's priority "winnable battles," public health priorities with large-scale impact on health and with known, effective strategies to address them (www.cdc.gov/WinnableBattles).

### Variations in Web-based health resource provision

We also observed a spectrum of college offerings of Web-based health resources. We found a high proportion of colleges with student health services and a related increased likelihood of the presence of online health resources. We also found that small and 2-year colleges were less likely than large and 4-year colleges to provide Web-based health resources, which we theorize relates to limited resources. Yet, most colleges in the United States are small, and approximately a quarter are 2-year colleges. With lower financial barriers to providing Web-based health resources online compared with on-campus health facilities, the use of the Internet may be a low-cost means for colleges to provide health promotion and prevention resources to young adults during their crucial time of health behavior development.

### Future of Web-based health resources for colleges

Nearly 60% of colleges in the US use Web-based resources to address health-related topics on their websites. The Internet is playing an increasing role in the interface between patients and providers in health promotion, disease prevention, and management. To our knowledge, this is the first study to characterize how US colleges use their institutional websites to improve the health of their students. Given that our analysis is based on a random sample of colleges, it should generalize to the broader group of all US colleges.

College websites represent a unique subset of websites with potential for high impact given the characteristics of college students. Although few best practices in Web-based health resources targeting college students exist, a few studies of college interactive Web-based health programs suggest significant potential, particularly in behavior modification around substance abuse (alcohol and tobacco) (24,25), sexually transmitted diseases (26), and mental health (27,28). While it appears that colleges with greater resources are more likely to provide Web-based health resources, partnerships with private groups to provide these resources, as described above, may be a practical way for more colleges to maximize their online presence. Further work needs to examine both the quality of Web-based health resource content and also the degree to which different types of college Web-based health resources actually influence health-related behaviors and outcomes.

### Limitations

We acknowledge several limitations to this study. First, our study assumes that college websites are static representations of available resources. However, because of the dynamic and fluid nature of websites, they may be incomplete, incorrect, or not up-to-date at the time they were accessed. Also, we did not assess the content of health information and quality of health resources. Finally, we tested several associations between institutional characteristics and provision of health resources, raising the possibility of chance findings existing with multiple testing.

### Conclusions

We found general widespread presence of Web-based health resources of various delivery modes on US college websites. Colleges with certain characteristics related to general resource availability were more likely to provide Web-based health resources. Most US adults spend some time in higher education, so colleges have a unique public health opportunity to provide important preventive health care information via the Internet. Although further research in this new modality is warranted, Web-based programs hold promise for reaching US college students.

## Figures and Tables

**Table 1.** Characteristics of Sampled US College Websites, February-April 2009

**Characteristic**	No. of Colleges (%)^a^
**All **	426 (100)
**2-Year colleges **	116 (38.6)
**Public colleges **	276 (55.7)
**College size**	
Small (<5,000 students)	130 (70.8)
Medium (5,000-9,999 students)	149 (14.0)
Large (≥10,000 students)	147 (15.1)
**College region**	
Northeast	109 (20.8)
South	105 (34.3)
Midwest	111 (25.2)
West	101 (19.7)
**Health professions school or program affiliation**	315 (62.8)
Nursing school or program	304 (61.1)
Medical school	38 (19.7)
School of public health	28 (11.1)
**Characteristics of student health services**	
Presence of student health or counseling services	371 (77.3)
Physician, nurse, or NP/PA listed on staff	270 (49.1)
At least 1 physician listed on staff	193 (31.5)
Mental health provider listed on staff	321 (65.6)
Health educator listed on staff	91 (10.9)
Nutritionist listed on staff	48 (6.0)

Abbreviation: NP/PA, nurse practitioner/physician assistant.

a Values are weighted to reflect the inverse sampling probability. On the basis of the full directory of US institutions of higher education, weights were created for the sampling strata based on the inverse sampling probability, such that the weighted results should approximate a representative national sample.

**Table 2:** Odds of Web-Based Health Resources, by Category and Institutional Characteristics, in Sampled Websites From US Colleges, February-April 2009

**Institution Characteristic**	Adjusted Odds Ratios (95% CI)^a^			

	**General Health**	**Reproductive and Sexual Health**	**Substance Abuse**	**Mental Health**
**Student health services**				
None	[Reference]			
Present	5.6 (1.4-23.1)	1.9 (0.4-10.0)	5.5 (0.7-41.4)	4.0 (0.9-18.6)
**Health educator^b^**				
None	[Reference]			
Present	7.8 (2.2-28.1)	5.2 (1.8-14.6)	3.9 (1.3-12.3)	3.6 (1.1-11.2)
**Health professions school affiliation^c^**				
None	[Reference]			
Present	1.0 (0.6-1.8)	1.1 (0.4-3.1)	1.1 (0.4-3.1)	1.1 (0.5-2.3)
**Type of college**				
2-year	[Reference]			
4-year	7.2 (2.4-22.3)	6.0 (2.5-14.1)	10.9 (3.5-33.7)	6.5 (4.1-10.4)
Private	[Reference]			
Public	1.5 (0.7-3.0)	1.6 (0.6-4.0)	1.5 (0.6-3.5)	1.6 (0.7-3.8)
**Size**				
Small (<5,000 students)	[Reference]			
Medium (5,000-9,999 students)	2.7 (1.1-6.7)	1.8 (0.6-5.7)	2.1 (1.0-4.6)	2.9 (1.7-5.0)
Large (≥10,000 students)	4.9 (1.0-23.7)	4.9 (1.0-24.0)	5.3 (2.1-13.1)	4.7 (2.8-8.0)

Abbreviation: CI, confidence interval.

a Each column represents separate logistic regression models that include all variables in the column and account for sampling weights. On the basis of the full directory of US institutions of higher education, weights were created for the sampling strata based on the inverse sampling probability, such that the weighted results should approximate a representative national sample.

b Defined as anyone listed on the website as a health educator; this could include those with degrees such as RN, NP, CHES, MPH, MA, or PhDs.

c Includes medical schools, nursing schools or programs, and public health schools or programs.

**Table 3.** Proportion of Sampled US College Websites With Health Information, by Category and Topic, February-April 2009

**Category^a^**	Weighted %^b^ (95% CI)
**General health information**	24.4 (16.1-32.7)
Asthma	1.6 (−0.4-3.6)
Cold and influenza	14.8 (10.1-19.5)
Meningitis	9.8 (6.5-13.1)
Methicillin-resistant *staphylococcus aureus *infection	4.3 (2.0-6.6)
Nutrition	7.0 (2.2-11.8)
Obesity	0.3 (−0.03-0.8)
Exercise or physical activity	4.2 (0.59-7.8)
General safety	3.8 (−0.4-8.0)
**Reproductive and sexual health information**	17.5 (8.3-26.7)
Birth control	4.4 (−0.6-9.3)
Cervical cancer screening (Papanicolaou test)	2.8 (−0.4-8.0)
HIV	5.3 (2.2-8.3)
Sexual assault	13.3 (6.7-20.0)
Sexually transmitted infections	7.1 (1.8-12.4)
**Substance abuse information**	15.0 (9.1-20.9)
Alcohol use	12.7 (8.2-17.2)
Drugs use	6.6 (3.8-9.3)
Tobacco use and cessation	6.8 (3.4-10.2)
Prescription drug abuse	1.8 (0.1-3.5)
**Mental health information**	18.2 (8.8-27.6)
Anxiety and stress	13.2 (6.9-19.5)
Depression and suicide	11.0 (3.8-18.2)
Eating disorders	8.7 (8.8-13.6)
Time management	3.0 (−0.2-6.2)
Wellness	4.6 (−0.2-9.4)

Abbreviation: CI, confidence interval.

a Subcategories in a major heading are not exclusive, and the subcategories do not add up to the major heading.

b Weights are based on inverse sampling probability. On the basis of the full directory of US institutions of higher education, weights were created for the sampling strata based on the inverse sampling probability, such that the weighted results should approximate a representative national sample.

## Appendices

### Appendix A. Website Survey Abstraction Tool

Instructions

There are 2 main categories you will be looking for:

A. Information on the college

1. Student health services staff
2. Affiliation with health profession schools

B. Web-based health resources in 4 topical areas

1. General health
2. Reproductive and sexual health
3. Substance use
4. Mental health

For the above (1, 2, 3, and 4) you will be looking for 3 different Web-based delivery modes for each topical area:

1. Health information
2. Outside Web links to health resources
3. Interactive Web-based health program

Where you will look for data:

1. Main college/university website
2. Institution's student health services
3. Counseling services' websites

You may search these websites in any order, but make sure to check all 3 for the content we are looking for. In order to get to the student health services, you can go to main college website (URL provided), then go to the student health services link if there is one, or search for it on the main college website. Counseling services may be within Student Health Services or a separate department.

Sources that we will NOT use:

- Student groups/activities websites
- Personal student Web pages

When abstracting data and looking for the desired content, you may use any technique, such as:

1. Following links (by clicking on them) within the site that you think will lead you there. For example, college website>> student health services>> health topics >> asthma
2. Using website search boxes, (eg, search term "asthma")
3. Looking at the sitemap of a website if there is one
4. Using the find function on a Web page (eg, find "asthma")

Cautions on data collection:

* Before entering data, please check website address and make sure that the site is part of the official college or university site (.edu), or if it is a link to an outside or commercial source, please document as such.

* Health information and programs must be geared towards students (undergraduate and graduate). Programs may include faculty and staff participation but should not be advertised as primarily or exclusively for faculty and staff.

* Do not enter data into shaded regions on spreadsheet.

0 = No, none — could not find any listings

1 = Yes

99 = Don't know, not sure — ambiguous

**A. Information on the college**

**1. Student health services (SHS):**

1aa – Is there a student health center?

- Go to SHS and look for a staff page. If no staff page, try to look in each department for number of providers.
- If staff is not listed individually, but it states that there are particular providers, do a minimum count of those that are listed (eg, if they say they have doctors and nurses, then count 1 for each).

1a – Is the staff named/listed?

If they are not listed individually, but a list of types of providers, do a minimum count of those that are listed.

For example, "We have pediatricians, orthopedists, nutritionists, and counselors." Then list 1a as 0, but 1b = 2, 1c = 1, 1f = 1.

1b – Count physicians listed (they must have 1 of the following degrees: MD, DO).

1c – Count counselors/psychiatrists/psychologists; you may need to go to counseling Web page (they could have 1 of the following degrees: PhD, PsyD, EdD, MA, MS, MD, DO).

1d – Count midlevel providers (they could have 1 of the following degrees: PA, NP, CRNP).

1e – Count registered nurses and medical assistants. If you cannot distinguish RN/Med technicians/assistants from midlevel providers, place them in RN category.

1f – Count nutritionist or registered dietician (RD).

1g – Count health educators; count anyone that they list as a health educator. Anyone with a CHES degree should be counted as a health educator. However, they may have other degrees such as RN or MA.

1h – Count physical activity trainers listed (may need to go to recreation services).

**Affiliation with other health professions programs or schools:**

- Identify colleges/universities with any affiliations with other health professions schools (nursing, medical, public health). This can include, but is not limited to the institution's own health professions schools.
- Specifically, you are looking for:
  1i — Nursing program or school
  1j — Medical school
  1k — Public health program or school
- You may either look at the institution's webpage for the listings of schools or promotion of an established relationship with health profession school or search for schools.
- Search terms such as "nursing school," "medical school," "public health school," on the institutions website search.
- Affiliation does not include broad lists of available research institutions where there are opportunities/projects for students to whom there is no formal affiliation.

0 = None — clearly none

1 = Listed on website

**B. Web-based health resources**

For each of the 4 health topics listed below, look for:

- Health information on various topics listed on SHS site
- Outside Web links
- Interactive Web-based health programs

- **Health information** is more than just listing services that are available around the topic/disease, but more detailed information about symptoms, diagnosis, treatment, etc. For example, describing the signs, symptoms, and treatment of chlamydia infection counts as health information. However, a website that only states that student health provides services for chlamydia does not count.
- **Outside Web links** to health resources are Internet links that take you from the college website to an outside (other-than-that-college) website that provides health information or health related resources.
- **Interactive Web-based health programs** are interactive programs accessed on the Internet

**2. General health information:**

2a – asthma

2b – common cold and influenza/flu, cough

2c – diabetes

2d – eating disorders — anorexia or bulimia

2e – HIV/HIV testing/screening

2f – meningitis

2g – methicillin-resistant *Staphlococcus aureus* infection

2h – general nutrition/eating well — this is general nutrition information that does not specifically focus on weight management/loss

2i – nutrition — weight loss focused — this is information that is explicitly or appears to be related to weight-loss or weight control

2j – overweight or obesity management — any information that addresses management or treatment of weight. Can be weight loss or weight management, must talk specifically about weight in context of being "overweight or obese," "excess weight," "heavy," "weighing more than you'd like."

2k – physical activity, fitness, exercise

2l – safety/violence/abuse

**3. Reproductive and sexual health information:**

3a – birth control

3b – cervical cancer screening/papanicolaou smear testing (Pap smear)

3c – HIV/AIDS (human immunodeficiency virus/acquired immunodeficiency syndrome) — if information available on HIV/AIDS, 2e should also be counted

3d – sexual assault — may also find in counseling services

3e – sexual health, sexually transmitted infections/diseases (STI/STD)

**4. Mental health information:**

4a – anxiety/stress management

4b – depression/suicide

4c – eating disorder — this is also listed in 2d so that it is not missed as it might be on either website. They are equivalent, and should be the same number

4d - Internet addiction

4e - time management

4f - wellness information

**5. Substance use information:**

5a – alcohol/drinking

5b – drug use/abuse/addiction — marijuana, heroin, cocaine

5c – smoking/tobacco/nicotine use or cessation

5d - prescription medication — safety, Rx drug abuse

Sample view of data collection spreadsheet

Reviewer #: _______________________

Date entered: _______________________

**Table:**

** **	**School Name (and Campus Campus if Relevant)**	College A			College B		
	**School number**	Prepopulated			Prepopulated		
	**Website address URL**	Prepopulated			Prepopulated		
	**City, state**	Prepopulated			Prepopulated		
	Region	Calculated			Calculated		
	**2-year or 4-year**	Prepopulated			Prepopulated		
	**Public/private**	Prepopulated			Prepopulated		
	**Student body size/student enrollment**	Prepopulated			Prepopulated		
	**Size**	Calculated			Calculated		
**A. Information on the college**							
**1.**	**Student health services:**						
1aa	Is there a student health center?						
1a	Is the staff named/listed?						
1b	No. of physicians listed						
1c	No. of counselors/ psychiatrists/psychologists						
1d	No. of midlevel providers (PA, NP)						
1e	No. of registered nurses and medical assistants						
1f	No. of nutritionist or registered dieticians						
1g	No. of health educators						
1h	No. of physical activity trainers						
	** Affiliation with other health professions programs/schools**						
1i	Nursing program or school						
1j	Medical school						
1k	Public health school						
**B. Web-based health resources**							
**2.**	**General health information:**	**Health information**	**Outside Web link**	**Interactive program**	**Health information**	**Outside Web link**	**Interactive program**
2a	Asthma						
2b	Common cold and influenza/flu, cough						
2c	Diabetes						
2d	Eating disorders — anorexia or bulimia						
2e	HIV/HIV testing/screening						
2f	Meningitis						
2g	MRSA infection						
2h	General nutrition/eating well						
2i	Nutrition — weight loss focused						
2j	Overweight or obesity management						
2k	Physical activity, fitness, exercise						
2l	Safety/violence/abuse						
**3.**	**Reproductive and sexual health information**	**Health information**	**Outside Web link**	**Interactive program**	**Health information**	**Outside Web link**	**Interactive program**
3a	Birth control						
3b	Cervical cancer screening/Pap smear						
3c	HIV/AIDS						
3d	Sexual assault						
3e	Sexual health, STI/STD						
**4.**	**Mental health information**	**Health information**	**Outside Web link**	**Interactive program**	**Health information**	**Outside Web link**	**Interactive program**
4a	Anxiety/stress management						
4b	Depression/suicide						
4c	Eating disorder						
4d	Internet addiction						
4e	Time management						
4f	Wellness information						
**5.**	**Substance use information**	**Health information**	**Outside Web link**	**Interactive program**	**Health information**	**Outside Web link**	**Interactive program**
5a	Alcohol/drinking						
5b	Drug use/abuse/addiction — marijuana, heroin, cocaine						
5c	Smoking/tobacco/nicotine use or cessation						
5d	Prescription medication — safety, Rx drug abuse						

Abbreviations: PA/NP, physician's assistant/nurse practitioner; MRSA, methicillin-resistant *Staphlococcus aureus* infection; STI/STD, sexually transmitted/sexually transmitted disease infection.

### Appendix B. List of Key Search Terms

**1. Student health services**

- staff
- providers
- doctors, physicians
- nurses, RN, medical assistants (MA)
- midlevel provider, physician assistant (PA), nurse practitioner (NP)
- counselors, psychiatrists, psychologist
- nutritionist, dietician
- health educator (can search degree CHES)
- physical trainers

**2. Affiliation with other health professions programs or schools**

- Nursing program or school
- Medical school
- Public health school

**3. General health information**

2a – Asthma, respiratory

2b– common cold, influenza, flu, cough

2c– diabetes

2d – eating disorders — anorexia, bulimia, binge, purge

2e – HIV/HIV testing/screening

2f – Meningitis

2g – Methicillin-resistant *Staphlococcus aureus* infection (MRSA)

2h – nutrition, eating well

2i – weight loss, weight control

2j – Overweight or obesity management, obese, excess weight

2k – physical activity, fitness, exercise

2l – safety, violence/abuse

**4. Reproductive and sexual health information**

3a – birth control, oral contraceptive, condom, the pill, depo

3b – cervical cancer screening/papanicolaou smear testing (Pap smear)

3c – HIV/AIDS (human immunodeficiency virus/acquired immunodeficiency syndrome)

3d – sexual assault — rape, date rape, harassment

3e – sexual health, sexually transmitted infections/diseases (STI/STD)

**5. Mental health information**

4a – anxiety, stress, stress management

4b – depression, depressed, suicide, feeling blue/down

4c - eating disorder

4d – Internet addiction

4e – time management

4f – wellness

**6. Substance use information**

5a – alcohol/drinking

5b – drug use/abuse/addiction — marijuana, heroin, cocaine

5c – smoking/tobacco/nicotine use or cessation

5d – prescription medication — safety, Rx drug abuse

Abbreviation: CHES, certified health education specialist.

## References

[1] (2009). School enrollment — social and economic characteristics of
students: October 2008.

[2] Arnett JJ (2000). Emerging adulthood — a theory of development from the late
teens through the twenties. Am Psychol.

[3] Lipnickey SC (1988). University students' knowledge and use of health
resources. Health Values.

[4] (2008). American College Health Association — National College Health
Assessment: reference group data report; Spring.

[5] Escoffery C, Miner KR, Adame DD, Butler S, McCormick L, Mendell E (2005). Internet use for health information among college
students. J Am Coll Health.

[6] Hansen DL, Derry HA, Resnick PJ, Richardson CR (2003). Adolescents searching for health information on the Internet: an
observational study. J Med Internet Res.

[7] Berland GK, Elliott MN, Morales LS, Algazy JI, Kravitz RL, Broder MS (2001). Health information on the Internet: accessibility, quality, and
readability in English and Spanish. JAMA.

[8] Hanauer D, Dibble E, Fortin J, Col NF (2004). Internet use among community college students: implications in
designing healthcare interventions. J Am Coll Health.

[9] Gray NJ, Klein JD, Noyce PR, Sesselberg TS, Cantrill JA (2005). Health information-seeking behaviour in adolescence: the place of
the Internet. Soc Sci Med.

[10] Skinner H, Biscope S, Poland B, Goldberg E (2003). How adolescents use technology for health information:
implications for health professionals from focus group
studies. J Med Internet Res.

[11] (2000). 21 Critical health objectives for adolescents and young adults.

[12] (2000). Healthy People 2010. 2nd edition. Understanding and improving health and
objectives for improving health. Vols. 1 and 2.

[13] Kisch J, Leino EV, Silverman MM (2005). Aspects of suicidal behavior, depression, and treatment in
college students: results from the spring 2000 national college health
assessment survey. Suicide Life Threat Behav.

[14] Zivin K, Eisenberg D, Gollust SE, Golberstein E (2009). Persistence of mental health problems and needs in a college
student population. J Affect Disord.

[15] Eisenberg D, Golberstein E, Gollust SE (2007). Help-seeking and access to mental health care in a university
student population. Med Care.

[16] Mokdad AH, Marks JS, Stroup DF, Gerberding JL (2004). Actual causes of death in the United States, 2000. JAMA.

[17] Johnston LD, O'Malley PM, Bachman JG, Schulenberg JE (2007). Trends on cigarette smoking and smokeless tobacco.

[18] Messer K, Trinidad DR, Al-Delaimy WK, Pierce JP (2008). Smoking cessation rates in the United States: a comparison of
young adult and older smokers. Am J Public Health.

[19] Centers for (2009). Cigarette smoking among adults and trends in smoking cessation
— United States, 2008. MMWR.

[20] Ogden CL, Carroll MD, Curtin LR, Lamb MM, Flegal KM (2010). Prevalence of high body mass index in US children and
adolescents, 2007-2008. JAMA.

[21] Williamson DF, Kahn HS, Remington PL, Anda RF (1990). The 10-year incidence of overweight and major weight gain in US
adults. Arch Intern Med.

[22] Sheehan TJ, DuBrava S, DeChello LM, Fang Z (2003). Rates of weight change for black and white Americans over a
twenty year period. Int J Obes Relat Metab Disord.

[23] Flegal KM, Carroll MD, Ogden CL, Curtin LR (2010). Prevalence and trends in obesity among US adults,
1999-2008. JAMA.

[24] Walters ST, Vader AM, Harris TR (2007). A controlled trial of web-based feedback for heavy drinking
college students. Prev Sci.

[25] An L, Klatt C, Perry CL, Lein EB, Hennrikus DJ, Pallonen UE (2008). The RealU online cessation intervention for college smokers: a
randomized controlled trial. Prev Med.

[26] Noar SM, Black HG, Pierce LB (2009). Efficacy of computer technology-based HIV prevention
interventions: a meta-analysis. AIDS.

[27] Chiauzzi E, Brevard J, Thum C, Decembrele S, Lord S (2008). MyStudentBody-Stress: an online stress management intervention
for college students. J Health Commun.

[28] Haas A, Koestner B, Rosenberg J, Moore D, Garlow SJ, Sedway J (2008). An interactive web-based method of outreach to college students
at risk for suicide. J Am Coll Health.

